# Pharmaceutical Dispersion Techniques for Dissolution and Bioavailability Enhancement of Poorly Water-Soluble Drugs

**DOI:** 10.3390/pharmaceutics10030074

**Published:** 2018-06-23

**Authors:** Xingwang Zhang, Huijie Xing, Yue Zhao, Zhiguo Ma

**Affiliations:** 1 Department of Pharmaceutics, College of Pharmacy, Jinan University, 601 West Huangpu Avenue, Guangzhou 510632, China; zhangxw@jnu.edu.cn; 2 Institute of Laboratory Animals, Jinan University, 601 West Huangpu Avenue, Guangzhou 510632, China; huijie920326@163.com (H.X.); tsukimoon@126.com (Y.Z.)

**Keywords:** poorly water-soluble drug, pharmaceutical dispersion, dissolution, bioavailability

## Abstract

Over the past decades, a large number of drugs as well as drug candidates with poor dissolution characteristics have been witnessed, which invokes great interest in enabling formulation of these active ingredients. Poorly water-soluble drugs, especially biopharmaceutical classification system (BCS) II ones, are preferably designed as oral dosage forms if the dissolution limit can be broken through. Minimizing a drug’s size is an effective means to increase its dissolution and hence the bioavailability, which can be achieved by specialized dispersion techniques. This article reviews the most commonly used dispersion techniques for pharmaceutical processing that can practically enhance the dissolution and bioavailability of poorly water-soluble drugs. Major interests focus on solid dispersion, lipid-based dispersion (nanoencapsulation), and liquisolid dispersion (drug solubilized in a non-volatile solvent and dispersed in suitable solid excipients for tableting or capsulizing), covering the formulation development, preparative technique and potential applications for oral drug delivery. Otherwise, some other techniques that can increase the dispersibility of a drug such as co-precipitation, concomitant crystallization and inclusion complexation are also discussed. Various dispersion techniques provide a productive platform for addressing the formulation challenge of poorly water-soluble drugs. Solid dispersion and liquisolid dispersion are most likely to be successful in developing oral dosage forms. Lipid-based dispersion represents a promising approach to surmounting the bioavailability of low-permeable drugs, though the technique needs to traverse the obstacle from liquid to solid transformation. Novel dispersion techniques are highly encouraged to develop for formulation of poorly water-soluble drugs.

## 1. Introduction

Drugs with poor aqueous solubility are still an ongoing challenge in the successful formulation of therapeutic products due to their low oral bioavailability. It is a hard nut to crack that has discouraged pharmaceutical practitioners for many years. In the 1990s; the biopharmaceutical classification system (BCS) was introduced to characterize various drugs according to their solubility and permeability [1]. It reports that over 70% of drugs and active entities are poorly water-soluble compounds (BCS II or BCS IV) due to the considerable involvement of high throughput screening and combinatorial chemistry [2]. These active pharmaceutical ingredients (APIs) often suffer from formulation challenges because of limited dissolution and/or low permeability. Accordingly; applicable formulation techniques are highly aspired to improve the apparent solubility or dissolution of poorly soluble drugs and thus enable them become bioavailable.

A variety of formulation strategies have been explored to overcome the poor aqueous solubility of drugs, including micronization [3], nanocrystalization [4], salification [5], cyclodextrin inclusion [6,7], cocrystallization [8], micelle solubilization [9], solid dispersion [10], liquisolid technique [11], and encapsulation in nanoparticles [12]. Generally, there are two methods to prepare nanodrug, namely top-down and bottom-up techniques. The former is a straightforward approach to reducing a drug’s size by the mechanical force (grinding or crushing); the latter is a simple and self-dispersion process where the drug is embedded or dissolved in carrier excipients/vehicles in molecular or amorphous state by solubilization or self-assembly [13]. Of course, recrystallization from molecular solution by antisolvent precipitation also represents a bottom-up technique for preparing drug nanocrystals [14]. However, the products produced by the top-down technique tend to result in broad size distribution and insufficient physical stability due to potential Ostwald ripening, which limits its potential application of this technique. The bottom-up technique, dispersion starting from molecules, almost can maximize the dispersion of a drug and lead to more stable dispersion systems (amorphous, molecular or colloidal). Therefore, the bottom-up dispersion technique represents the most promising approach for pharmaceutical processing.

Pharmaceutical engineering involves all sorts of dispersion systems, including suspension system, colloidal system and solution system, in which a drug can be dispersed by itself or in a solid matter, a semisolid matter, a solvent or nanoparticles. Among these, solid dispersion (SDs), lipid-based dispersion and liquisolid dispersion are well-developed and more commonly used pharmaceutical dispersion techniques. These dispersion systems have been widely applied to formulation of poorly water-soluble drugs to address the issues related to solubility and permeability. Solid dispersion technology is a method of dispersing a drug in an inert carrier excipient (normally a water-soluble polymer) in the solid form. This technique allows complete removal of drug crystallinity and molecular dispersion of a poorly soluble drug in a hydrophilic polymeric carrier [15]. In solid dispersions, the specific surface area of drug is dramatically amplified and the drug is always in high-energy state, which greatly facilitates the dissolution. The performance of SDs as bioavailability enhancer has been widely evaluated for different drugs with encouraging results [16]. Lipid-based formulations are a series of preparations consisting of oils or lipids as basic excipients [17], in which the drug is highly dispersed or completely solubilized in the lipophilic core. In addition to providing a supersaturated drug concentration in the gastrointestinal tract, lipid formulations have the advantages of motivating intestinal lymphatic drug transport and optimizing enterocyte-based drug transport and disposition whereby to reduce the first-pass effect and increase the lipophilic drug’s absorption [18]. The potential utility of lipid formulations as a means of bioavailability enhancer for poorly water-soluble drugs has been critically reviewed [19,20]. 

Liquisolid system as a viable alternative to the conventionally used dispersion techniques for dissolution and bioavailability improvement has received considerable attention in recent years [21]. Liquisolid formulations involve liquid medication in solid powdered form that possess a drug delivery mechanism similar to soft capsules [22]. In the liquisolid systems, a drug is completely dissolved in a non-volatile solvent and molecularly dispersed in suitable carrier and coating materials. Liquisolid system breaks away from the constraint of Van der Waals’ attraction and hydrophobic interaction between drug particulates, thus presenting the highest dispersibility and physical stability among various dispersion systems. Better bioavailability for an orally administered poorly water-soluble drug can be achieved through a liquisolid formulation since the drug is already in solution. Of course, other dispersion techniques are also in active development, such as co-precipitation, concomitant crystallization and inclusion complexation. These techniques provide flexible options to tackle the low bioavailability of “problem” drugs caused by poor aqueous solubility.

In this article, a comprehensive survey on the use of solid dispersion, lipid-based solubilization and liquisolid technique for dissolution and bioavailability enhancement of poor water-soluble drugs are carried out. Specific aspects focus on the formulation development, excipient application, preparative techniques and oral delivery efficiency of these dispersion-based formulations. In addition, other dispersion techniques that can improve the dissolution as well as bioavailability are also briefly outlined. It is strongly recommended that these promising pharmaceutical dispersion techniques can well serve for the development of oral dosage forms of poorly water-soluble drugs.

## 2. Interrelation between Dispersion and Drug Dissolution/Absorption

**Figure 1:**
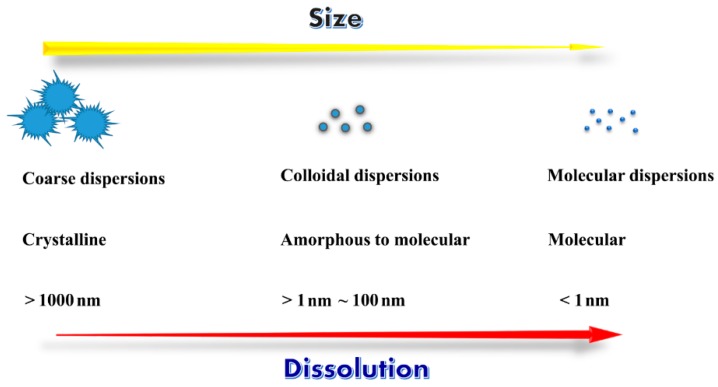
Dimension and physical properties of different kinds of dispersions.

Dispersion is a technique resulting in a substance dispersed or embedded in another molecule or continuous phase. A dispersion can be classified in a number of ways according to the size and the state of dispersed matter. Generally, there are three main types of dispersions as depicted in Figure 1: coarse dispersions (suspensions); colloidal dispersions (nanoparticles); and molecular dispersions (true solution, liquid or solid state). The term “dispersion” does not involve covalent bonds, and instead generates a reversible agglomerate containing two or more substances by van der Waals forces, hydrogen bond, hydrophobic interaction and/or physical entanglement [23]. Dispersing a drug in another material is an effective means to overcome the intermolecular force between drug molecules and realize a quick dissolution. 

Drug dissolution refers to a process that the interaction between drug molecules is displaced by the one between the drug and dissolution medium [24]. If the intermolecular force between drug molecules is prematurely minimized, the dissolution will be significantly speeded up. Weak interactions between drug and carrier formed in dispersions not only well maintain the dispersion state of a drug in a carrier, but also produce a higher internal energy between drug and carrier than between drug molecules. This high-energy state greatly contributes to the drug dissolution. Drug dissolution from formulations is particularly important for those drugs with a short absorption window, since they might have passed their absorptive sites by the time they have dissolved. Dissolution is the prerequisite of drug absorption by the gastrointestinal epithelia. Low aqueous solubility always results in a slow drug dissolution rate in the coarse dispersion systems. Formulating poorly soluble drugs into ultrafine dispersions can lower the energy barrier for dissolution in advance and thus enhance the dissolution rate. For BCS II drugs, it is a feasible way to promote the oral absorption by preparing dissolution-unconfined dispersions. However, for BCS IV drugs, it is not enough to improve the absorption extent utilizing a dispersion strategy that merely overcomes the dissolution limit [25]. It must simultaneously surmount the dissolution and absorption barriers. Formulation techniques that have the function of dispersion plus absorption-promoting effect are intrinsically required to develop. In this respect, lipid-based formulations have demonstrated great potential in absorption betterment due to high biocompatibility and interaction with the cell membrane [26]. Of course, lipid dispersions, often in the form of nanoparticles, do not have to experience a dissolution process for subsequent absorption. Anyhow, formulating a poorly soluble drug into eligible dispersions can create favorable conditions for its dissolution and absorption.

## 3. Solid Dispersion Technique

### 3.1. Solid Dispersions

**Figure 2:**
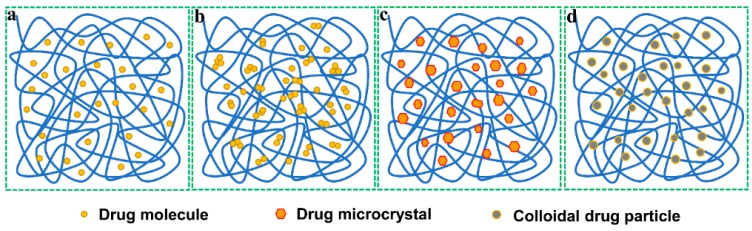
Physical state of drug in solid dispersions: (**a**) molecular state, forming solid solution; (**b**) amorphous state, forming amorphous dispersions; (**c**) microcrystal state, forming simple dispersions; (**d**) colloidal state, forming colloidal dispersions.

Solid dispersions are a dispersion mixture of one or more active ingredients in an inert carrier at the solid state prepared by melting, solvent, solvent-melting or other methods. The approaches used for preparing SDs are referred as solid dispersion techniques. According to Noyes–Whitney equation, the dissolution rate of a drug in a given medium depends on the concentration difference between the dissolving interface and the bulk solution. For poorly water-soluble drugs, the dissolving rate on the interface is positively associated with the particle size of drug, especially above 100 nm [27]. SDs can maximize the reduction of a drug‘s size by dispersing it in a large quantity of carrier excipient, thus increasing the absorption area, hence the bioavailability. In SDs, the drug can be in presence as molecular, amorphous, microcrystal or colloidal state (Figure 2), which is dependent on the formulation and preparative process thereof. The high-energy or metastable state of drug in SDs makes it tend to dissolve in a medium, as opposed to the bulk drug. Apart from drug solubilization, SDs can also improve the gastrointestinal absorption of poorly soluble drugs by affecting the absorptive epithelia, in particular those surfactant-based and absorption enhancer-containing SDs. Currently, the scale-up manufacturing of SDs has no longer been a limitation factor that hinders their success to the clinical application. SDs can either serve as a pharmaceutical intermediate used for preparation of various dosage forms such as tablets, capsules and granules, or as a final pharmaceutical product, e.g., pellets produced by one-step granulation in fluidized bed. During the past decades, there more than ten commercial SDs-based products have been marketed [10]. SDs are presenting an inspiring vision to solve the dissolution and bioavailability issues of poorly soluble drugs. Solid dispersion technique is more suitable for those drugs with low viscosity, less hygroscopicity and high glass transition temperature.

### 3.2. Carrier Excipients of SDs

Carrier excipients play an essential role in formation of SDs, drug dissolution and absorption, and stability of SDs. Pharmaceutical excipients that have been used for production of SDs are exhaustively collected in Table 1. The carrier excipients of SDs are generally classified into low-molecular-weight carriers, polymeric carriers and surfactant carriers [28]. They are highly water-soluble or hydrophilic in nature in the case of poorly water-soluble drugs. In physical property, low-molecular-weight carriers are generally crystalline (e.g., saccharides), amphiphilic copolymer carriers are semi-crystalline (e.g., Poloxamer), and homopolymer carriers are amorphous, such as polyethylene glycol (PEG) and polyvidone (PVP). In the early development of SDs, low-molecular-weight carriers were tentatively used, such as urea, saccharides and organic acids. These carrier excipients have high requirements for drug and solvent used. Moreover, the resulting SDs tend to become aging and unstable. In some cases, the low-molecular-weight compounds such as glucose and lactose negatively affect the gastrointestinal absorption of API, since the body preferably takes up the nutrients rather than the non-nutritive excipients. Compared to low-molecular-weight carriers, polymeric carriers possess larger molecular weight that can afford higher dispersibility and stronger recrystallization inhibition for drugs. For this end, polymeric carriers are currently widely used for the preparation of SDs, such as PEG, PVP and hydroxypropylmethylcellulose (HPMC). Nevertheless, the high viscosity, plasticity and hygroscopicity associated with macromolecules that make problems for production compromise their application in SDs. Polymeric carriers, not including surfactants, have inadequate absorption-promoting effect for poorly permeable drugs, which just provide necessary dispersibility. Carriers that possess a surfactant property, beyond dispersion powder, have the advantage of increasing drug absorption through interaction with the absorptive epithelia and inhibiting drug efflux transporters. To improve the performance of polymeric carriers, copolymers and functionalized polymers (e.g., PEGylated polymers) are developed for SDs. These novel carrier excipients are provided with excellent amphiphilicity, formability, solubilization or absorption-promoting characteristics. Examples include fatty acid macrogolglycerides (e.g., Gelucire 44/14 and Gelucire 50/13), poly(vinylpyrrolidone-*co*-vinyl acetate) (PVP/VA), and poly(vinyl acetate-*co*-vinyl caprolactame-*co*-ethylene glycol) (Soluplus^®^). 

Gelucire 44/14 and Gelucire 50/13 are non-ionic water dispersible surfactants composed of well-characterized PEG-esters, a small glyceride fraction and free PEG, which are able to self-emulsify upon contact with aqueous media, forming a fine dispersion [64]. The surface activity of such PEGylated carriers can improve the solubility and wettability of API in vitro and in vivo. Enhanced bioavailability was reported to be related with strong inhibition of P-glycoprotein efflux and metabolizing enzyme CYP3A4 [65]. Furthermore, this kind of carriers have good thermoplasticity suitable for use in the melt process. PVP/VA copolymers possesses proper flexibility, bioadhesion, water remoistenability and hardness, and have found usage in SDs as carrier excipient [60]. In comparison with PVP, PVP/VA has a lower hygroscopicity but higher bioadhesion, conferring it easy processing and good absorption-promoting capacity. These advantages make it more suitable for preparation of SDs for oral drug delivery. Soluplus^®^, a polyethylene glycol, polyvinyl acetate and polyvinylcaprolactame-based graft copolymer, has been extensively investigated for preparation of SDs [62,63]. Soluplus^®^ is a transparent solid excipient and can form solid solutions with many drugs [66]. Soluplus^®^ is an innovative excipient that enables new levels of solubility and bioavailability for poorly soluble APIs. Soluplus^®^ shows superior performance in forming solid solutions in the hot melt extrusion process thanks to its high flowability and excellent extrudability. The resulting solid solution makes API available in a dissolved state, resulting in significantly enhanced bioavailability in vivo. The products above-mentioned represents the latest development in carrier excipients of SDs, which will become the dominant excipients for production of SDs in the future [67]. 

### 3.3. Preparative Processes of SDs

**Figure 3:**
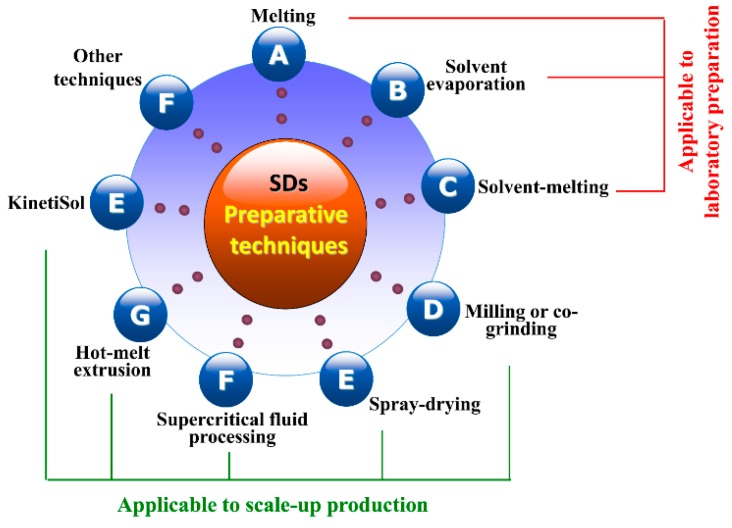
Commonly used preparative techniques for solid dispersions.

There are many kinds of techniques available for the production of SDs (Figure 3) [67], including melting, solvent evaporation, solvent-melting, milling, spray-drying, hot-melt extrusion, supercritical fluid processing, KinetiSol^®^, etc. Among these, some are applicable to scale-up production of SDs, while others just can be implemented in the laboratory. In the following section, we give a brief discussion on a variety of preparative processes involved in SDs.

#### 3.3.1. Melting Method

Melting method is the earliest technique used for SDs preparation proposed by Sekiguchi and Obi in 1961. They prepared sulfathiazole SDs by melting the drug with urea and investigated the drug absorption in the form of eutectic mixture [68]. In this process, drug and carrier are mixed evenly and then heated to make all components molten. Subsequently, the molten materials are subjected to quenching against a cold plate to obtain the congealed mass. Melting method is a straightforward and solvent-free process that is suitable for heat-stable drugs. But, this technique possesses some shortfalls such as high-temperature operation and difficulty in the later processing (e.g., product harvesting and pulverization) that limits its success in industrial production. Nevertheless, melting method can yet be regarded as a convenient preparative process for preliminary survey on the suitability of solid dispersion if the candidate is thermostable. For example, Alhayali et al. prepared ezetimibe/PVP/Poloxamer SDs by melt-quenching method [69]. The resulting SDs were more soluble than that produced by spray-drying.

#### 3.3.2. Solvent Evaporation Method

Solvent evaporation method circumvents the suffering of high temperature, thus suitable for heat-labile APIs. In this process, both drug and carrier are dissolved in organic solvent. After dissolving, the solvent is evaporated using a special apparatus, rotatory evaporator. The solid mass is ground, sieved and dried. It requires that the drug and selected carrier excipient can completely dissolve in the solvent so as to form amorphous dispersions or solid solution. Of course, this approach likewise confronts challenges from two aspects: to remove the residual solvent and to harvest the SDs. Ethanol is the proposed solvent to use in the production of SDs. But, in some cases, other harmful solvents have to be adopted in order to dissolve some water-insoluble drugs. If the issues of solvent residue and product harvesting in the evaporator can be overcome, solvent evaporation method may after all be accepted as workable technology for manufacturing SDs. With this technique, Dos Santos et al. developed SDs of β-lapachone using PEG 6000 and PVP k30 as hydrophilic polymers and evaluated the dissolution rate in aqueous medium [70].

#### 3.3.3. Solvent-Melting Method

Solvent-melting method is an improvement to melting method and solvent evaporation method. In this process, API is first dissolved in a small quantity of solvent and then added into the molten carrier excipient. The solvent used can be removed through instant evaporation upon contacting with the hot carrier or through an evaporator. This technique overcomes the disadvantages of high temperature and the use of larger volume of solvent, which is suitable for moderately thermostable and practically insoluble APIs. However, it also requires the therapeutic dose of API to be low. For instance, Chen et al. prepared emulsified SDs containing docetaxel by three approaches, i.e., melting method, solvent-melting method and solvent method [71]. It was shown that the dissolution of docetaxel from SDs prepared by the solvent-melting method was higher than that prepared by the melting method.

#### 3.3.4. Milling Method

Milling method, also known as co-grinding method, refers to a preparative process of SDs by exploiting external mechanical power to knead the drug and carrier together. The drug and carrier are physically mixed for some time in a blender and then charged into the chamber of a colloid mill or fluid-energy mill to grind strongly with or without a small amount of solvent. The resulting dispersions are collected, dried and pulverized ready for use. As an example, chlordiazepoxide and mannitol SDs were produced by this method [72]. Of note, SDs prepared by this technique generally exhibit inferior dissolution improvement than does the solvent evaporation or melting processes. Nevertheless, this technique is more suitable for scale-up production of SDs, which can be easily carried out using a grind machine.

#### 3.3.5. Spray-Drying Method

Spray-drying method refers to the preparation of SDs by dissolving or suspending the drug and carrier in a common solvent or solvent mixture and then drying it in a hot air stream to remove the solvent, which can be deemed as an improvement on the solvent method. Upon atomization, the solvent promptly evaporates and SDs are formed simultaneously. Spray-drying method can directly obtain SDs powders with good flowability or pellets by co-precipitating on the surface of blank beads using fluid-bed coating. It can completely yield drugs in the amorphous or molecular state [23], though sometimes drug crystallization partially takes place during the processing [73]. Spray-drying method provides a high feasibility for scale-up production of SDs using spraying dryers, especially using a fluidized equipment. 

#### 3.3.6. Supercritical Fluid Processing

Supercritical fluid (SCF) technology shows tremendous advantages and favorable operating conditions (low temperature and high dissolving power), making the method more attractive for SDs production. The most commonly used SCF for a variety of pharmaceutical applications is supercritical carbon dioxide (CO_2_). Apart from lower critical temperature (*T*_c_ = 31.1 °C) and pressure (*P*_c_ = 73.8 bar), CO_2_ is nontoxic, nonflammable and inexpensive for use. In general, two basic SCF techniques can be utilized to prepare SDs [74], namely rapid expansion of supercritical solution (RESS) and gas antisolvent precipitation (GAS). In RESS technique, a solution containing drug and carrier in the supercritical state is expanded rapidly through a nozzle. Due to rapid changes in density and solvent power, the solution becomes highly supersaturated and consequently SDs are immediately formed [75]. In GAS technique, drug and carrier are first dissolved in an organic solvent in a vessel. The solution is then pressurized with a supercritical fluid, resulting in precipitation of the solid as a fine powder upon solvent extraction by SCF [76]. Yin et al. prepared itraconazole SDs with HPMC, Pluronic F-127 and L-ascorbic acid using GAS in an attempt to enhance its dissolution and bioavailability [77]. Powder X-ray diffraction and Fourier transform infrared spectra indicated that itraconazole existed as an amorphous state in SDs. SCF technology provides a novel alternative approach to preparing SDs with high surface area, excellent flowability property and low solvent residue. This technology is equally applicable for scale-up production of SDs and can avoid most of the drawbacks associated with the routine methods.

#### 3.3.7. Hot-Melt Extrusion

Hot-melt extrusion is a process of applying heat and pressure to melt a polymer or mixture and force it through an orifice in a continuous process, which was introduced into the pharmaceutical field for SDs manufacturing in 1980s. The drug/carrier mixture is simultaneously melted, homogenized and then extruded with a twin-screw extruder. The resulting intermediates can be further processed into conventional dosage forms, such as tablets and capsules. The prominent advantage of hot-melt extrusion lies in the shorter subjection to high temperature, approximately for 1~2 min, which secures APIs that are somewhat heat-labile. Hot-melt extrusion has been successfully explored for the preparation of Soluplus^®^/artemisinin SDs [63]. Although artemisinin is a thermolabile drug, it can be processed under 110 °C to produce the SDs. Hot-melt extrusion can be regarded as an innovation toward the melting method, which reduces the difficulty of follow-up processing of SDs and is suitable for mass production likewise.

#### 3.3.8. KinetiSol^®^ Technique

KinetiSol^®^ technology represents a new processing paradigm for amorphous SDs, which can satisfy some unmet needs. Poorly soluble drugs that have high melting point and low solubility in organic solvents are becoming commonplace. KinetiSol^®^ dispersing is a new fusion-based process that has been developed to rapidly form SDs by exerting high shear and friction force without external heat input [78]. It can be conducted in a custom built compounder designed for pharmaceutical processing. The unit consists of a product containment vessel with a rotating shaft that has several blades facing outward from it. During operation, the blades rotate at a high velocity that rapidly processes the materials through the heat developed by shear and frictional motion of product within the vessel. The fusion mode is unique among various possessing technologies of SDs where no external energy input is required. This thermo-kinetic mixing is termed as KinetiSol^®^ dispersing. KinetiSol^®^ dispersing rapidly transfers drug and polymer blends into a molten state that thoroughly mixes the API with selected carrier excipient on a molecular level to achieve a single-phase amorphous system. The real-time temperature of dispersions is monitored by a computer-controlled module. Once reaching the designated end point, the molten material is immediately ejected from the unit. The total processing time is generally less than 20 s, and elevated temperature is typically not more than 5 s before discharge and cooling. KinetiSol^®^ provides technical supplement to the hot-melt extrusion and spray-drying processes when the API is thermally labile or unstable in organic solution. For example, LaFountaine et al. utilized this technique to formulate ritonavir, a drug with thermal, rheological and soluble limitations, into amorphous SDs using polyvinyl alcohol as carrier and confirmed the feasibility of this processing for production of SDs [79]. KinetiSol^®^ dispersing can be operated semi-continuously in a custom built device with the product throughput up to 1000 kg/h. Therefore, the technique is incredibly applicable to the commercial processing of SDs.

### 3.4. SDs-Based Dissolution and Bioavailability Enhancement

For poorly water-soluble drugs, especially BCS II ones, the limited step of gastrointestinal absorption is the dissolution process of drugs from their preparations. According to Noyes-Whitney equation, the dissolution rate is proportional to the surface area of dissolution. Reducing the particle size or enhancing the dispersibility of drug is an effective means to increase the surface area of dissolution. Solid dispersion technique not only can enhance the drug dispersibility, hence the surface area, but also can result in a high-energy state of drug (e.g., amorphous, molecular or colloidal crystal state) that largely facilitates the drug dissolution. In addition, some carrier excipients such as Poloxamer and Gelucire have the abilities of promoting drug absorption and inhibiting drug efflux. These advantages impart SDs excellent performances for oral delivery of various therapeutic agents [80].

Piperine, an alkaloid with poor water solubility, has been prepared into SDs with sorbitol, PEG and PVP by solvent evaporation method [36]. Piperine SDs with three different kinds of carriers all exhibited superior performance for enhancement of dissolution compared to physical mixtures and pure piperine. The transformation from crystalline to amorphous form as well as the assistance of hydrophilic carriers was assumed responsible for dissolution improvement. Deng et al. developed SDs formulations using Pluronic F68 and PEG as carriers to enable the oral delivery of α-asarone, a phytomedicine with poor solubility and bioavailability [81]. SDs prepared using hydrophilic polymers significantly enhanced the dissolution in vitro and oral bioavailability in vivo of α-asarone, showing a great potential for developing oral dosage form of α-asarone. In another example, SDs formulations consisting of itraconazole and Soluplus® were produced by hot-melt extrusion [82]. Higher maximum plasma concentration (*C*_max_) and area under plasma concentration-time curve (AUC) were achieved through SDs after oral administration compared to the levels resulting from a marketed product (Sporanox^®^). Efonidipine hydrochloride ethanolate (NZ-105) is a novel API with calcium antagonist activity, but has a very low solubility in water. Otsuka et al. employed the microwave technology, a modified melting method, to prepare NZ-105 SDs using hydroxypropyl methylcellulose acetate succinate as a carrier and urea as an auxiliary component [83]. It was showed that SDs prepared through such technique resulted in eightfold improvement in oral bioavailability compared with NZ-105 alone in beagle dogs. These cases indicated that SDs as a dosage form or intermediate have become a viable option for addressing the dissolution and bioavailability issues of poorly water-soluble drugs.

## 4. Lipid Dispersion Technique

Lipid dispersion refers to formation of nanoparticles using lipid excipients. Distinct from solid dispersion, the products resulting from lipid excipients are generally in the form of liquid dispersion. Even though solidified by spray-drying or lyophilization, they possess poor powder property, low storage stability, and great difficulty to pulverize. Nonetheless, formulating drug into lipid carriers represents an effective dispersion technique that can enhance the dispersibility of drug and create a supersaturated concentration in the gastrointestinal lumen for drug absorption. Herein, it is obviously inappropriate using the terms of solubility and dissolution to describe the dissolution characteristics of lipid-formulated drugs. Solubilization and release turn into the descriptors to characterize the drug lipid dispersions. Lipid dispersion technique is more applicable for formulation of highly lipophilic, low-melting-point, and poorly permeable drugs. These drugs are easily prepared into lipid nanoparticles with satisfactory physiochemical stability.

### 4.1. Lipid Dispersions Accomplished by Lipid Nanoparticles

**Figure 4:**
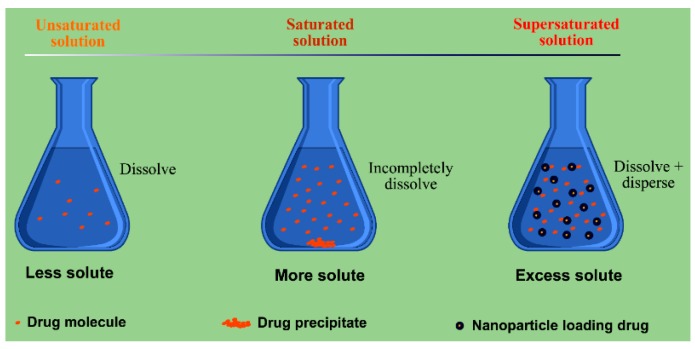
Schematic illustration of supersaturated state of drug in nanoparticle dispersion system.

Dispersion of drug can either be accomplished through excipient-free top-down (milling) and bottom-up (recrystallizing) methods that forms drug nanocrystals [84], or through excipient-assisted nanosizing that forms nanoparticles [85]. The drug dissolves or disperses in the hydrophobic excipients followed by being formulated into a variety of lipid-based nano-formulations. In general, the hydrophobic excipients are lipids or amphiphilic materials containing lipid moiety. A liquid formulation containing nanoparticles or nanocarriers is normally termed as “nanosuspensions”, too. From the viewpoint of dispersion, the nanosuspensions resemble the drug nanocrystals where the difference is only that the drug is dispersed in the lipid core rather than in the bulk solution. On the other hand, nanosuspensions can also be transferred into the solid modality by dehydration. The solidified or dried products are the same as SDs made from polymers with respect to dispersibility. Poorly water soluble drugs can completely form highly dispersed lipid dispersions with suitable excipients. However, lipid dispersions both in the form of nanosuspensions and lyophilized state are different from SDs with respect to drug release, absorption feature, processibility, usage and storage. The most predominant difference is the drug transport process where drug release does not have to take place for lipid dispersions and the active ingredient can be assimilated through nanocarriers or reconstituted colloidal particles [86]. SDs accelerate the drug dissolution and absorption by improving the apparent solubility and dissolving rate of drug, while lipid dispersions augment the surface area for drug absorption by providing supersaturated drug concentration in the gastrointestinal lumen (Figure 4). Lipid dispersion and solid dispersion achieve the same goal by different means. Lipid-based formulations provide a straightforward and ready-to-use dispersion for drug absorption that undergoes no disintegration and dissolution processes.

### 4.2. Commonly Used Lipid Dispersion Systems

#### 4.2.1. Solid Lipid Nanoparticles

Solid lipid nanoparticles (SLNs) represent the first generation of lipid nanoparticles composed of a high-melting-point solid lipid and a small number of surfactant, which are developed on the base of from O/W emulsions [20]. SLNs present a solid state both at room and body temperature, thus possessing high physical stability. They show multiple advantages as drug delivery system, such as high drug loading, sustaining drug release, facilitating drug absorption, and ease of scale-up production [87]. These features of SLNs make them more suitable for formulating poorly water-soluble drugs to ameliorate their oral bioavailability. High-pressure homogenization (HPH) is the most frequently used technique for the production of SLNs [88]. SLNs have become as a potential enhancer of bioavailability for various poorly soluble drugs [26].

#### 4.2.2. Nanostructured Lipid Carriers

SLNs exclusively involve solid lipids that have a high crystallinity in the lipid core, thus existing potential drug expulsion upon storage [89]. The solid lipids typically exhibit low capacity of dissolving poorly soluble drugs compared with liquid lipids. In this context, nanostructured lipid carriers (NLCs) are invented to overcome the limitations associated with SLNs owing to the highly ordered structure. NLCs are derived from SLNs by incorporating spatially incompatible liquid lipid into the solid core. The participation of liquid lipid creates an imperfect crystal matrix, resulting in higher drug loading and drug/lipid compatibility [90]. Depending on superb solubilizing and dispersing capacities, NLCs have turned into a promising nanocarrier and have been widely investigated for oral drug delivery [91].

#### 4.2.3. Nanoemulsions

Pharmaceutical nanoemulsions are generally O/W emulsions in the nanometer scale made up of oil phase, water phase, emulsifier, and a selected co-emulsifier. By virtue of facile preparation and smaller particle size, nanoemulsions have been getting considerable attention in recent years as smart drug delivery system [92]. The oil used in the nanoemulsions formulation is a liquid lipid, which provides a great practicality for high load of poorly soluble drugs. Nanoemulsions can spontaneously form in the presence of massive surfactants (~20% of the oil phase, *w*/*w*) [93]. The excellence in particle size (<100 nm) renders nanoemulsions a high dispersibility for drug and a great surface area for absorption. Nanoemulsions can either be water-containing formulation or water-free formulation. An example as water-free formulation is the self-microemulsifying drug delivery system (SEDDS), generally being developed into the dosage form of soft capsules [94]. Drug dispersing (molecularly dissolving) in the oil phase of nanoemulsions is equivalent to dispersing in the solid carrier. They just appear in different state and dispersibility, but have no substantial distinction. Therefore, nanoemulsions containing liquid lipid (oil) are also a kind of efficient dispersing vehicle.

#### 4.2.4. Liposomes and Phytosomes

Liposomes are spherical vesicles consisting of one or more bilayers formed by phospholipid and cholesterol. Due to flexible controllability and good biocompatibility, liposomes have become a talented drug delivery system, and several liposomal products are clinically available at present [95]. Liposomes not only can encapsulate hydrophilic molecules but also encapsulate hydrophobic molecules [96,97,98,99], thanks to holding both aqueous cavity and hydrophobic bilayer in structure. Among various preparative processes of liposomes, the film hydration method is the commonly used and more mature technique. In the process of lipid film formation, the drug is utterly dispersed in the lipid mixture. Liposomes as oral delivery vehicle have been extensively explored for a variety of active therapeuticals and have shown a huge potential for enhancement of bioavailability [100].

Unlike liposomes, phytosomes are not vesicle-based drug carrier. They are phospholipid dispersions containing a natural active ingredient [101]. Phytosomes are chemically conjugated drug-phospholipid complexes prepared by reacting a botanical active ingredient with phospholipid in an opportune solvent, which can be considered as novel entities [102]. The plant extracts or its monomers are firmly bound to phosphatidylcholine, a main constituent of phospholipids, resulting in a lipid compatible molecular complex. Phytosomes can significantly improve the pharmacokinetic and pharmacodynamic profiles of phytomedicines compared to the unmodified modalities [103]. Phytosomes are originally developed for handling the water-soluble phytomedicines with poor oral absorption due to their large molecular size and lack of lipophicity, but now not limited to water-soluble active compounds. Some highly hydrophobic molecules have been successfully formulated into phytosomes, such as silymarin [104], curcumin [105], and apigenin [106]. Phytosome technology has been widely utilized to potentiate various active ingredients including phytomedicines and chemical drugs, and has proven to be a useful tool for strengthening the potency of poorly water-soluble drugs.

### 4.3. Lipid Excipients

Lipids are substances consisting of fatty acids and their derivatives, including oils, fats, waxes, sterols, monoglycerides, diglycerides, triglycerides, and phospholipids. Lipid-based drug delivery systems are mostly constructed upon lipid vesicles or matrixes. The lipid excipients have abilities to solubilize, disperse, encapsulate and stabilize lipophilic drugs that are poorly water-soluble in nature, thus enhancing their bioavailability. The unique characteristics of lipid excipients have been motivating the pharmaceutical practitioners to develop various lipid-based formulations for coping with challenges from compounds with inadequate solubility and permeability [107]. Apart from the true solution and simple dosage forms, almost all colloidal dispersion systems are largely dependent on the use of lipid excipients. For instance, in the formulation of emulsions, the hydrophilic drugs are solubilized in the inner oil phase of emulsion droplets. In the case of liposomes, the hydrophilic drugs are entrapped into the lipid bilayer of vesicles. In SLNs and NLCs, the hydrophilic drugs are dissolved or dispersed in the lipid core of nanoparticles. For phytosomes, the natural compounds are physically coupled or chemically conjugated with the phospholipids. Lipid excipients are generally inert, in vivo biodegradable and biocompatible with the body, thus possessing high safety and accessibility for drug delivery. Many kind of lipid excipients have been approved by the regulatory agency (Food and Drug Administration, FDA) for use in pharmaceutical products [108]. Meanwhile, severe adverse reactions have not been reported on lipid excipients or formulations as yet. Table 2 lists the commonly used lipid excipients at large that are involved in the lipid-based formulations. With the advancement of excipients, it will usher in the rapid development of lipid-based formulations to revitalize poorly soluble and/or permeable drugs.

### 4.4. Lipid Nanocarriers-Based Enhancement of Bioavailability

The merits of lipid-based formulations for oral drug delivery have been profoundly reviewed [2,107,170]. Lipid-based formulations, in most cases, are colloidal dispersion systems that circumvent the rate-limiting steps of drug absorption related to conventional solid dosage forms, such as tablets, capsules and granules. It does not require the formulations to disintegrate and dissolve for drug absorption. Lipid-based formulations in the form of nanosuspensions provide a sufficiently large surface for drug absorption in the gastrointestinal gut. Even being digested, they can also result in a supersaturated drug concentration by way of reconstitution into micelles [171]. These characteristics create favorable conditions for drug absorption. Oral bioavailability of lipophilic drugs such as itraconazole can be improved even though they are co-administered with a fat-rich meal or vegetable oil. Lipid nanocarriers have positive effects on drug absorption by making supersaturated drug concentration, preventing drug precipitation, enhancing membrane permeability, inhibiting efflux transporters, reducing CYP enzymes, providing bioadhesion to the absorptive epithelia, stimulating secretion of chylomicrons and improving lymphatic transport [172]. As a result of lipid dispersion, it greatly augments the absorption rate and extent of lipophilic drugs. Oral route is the most preferred option that patients take a medication. Taking full advantage of the potential of lipid nanocarriers will enable some drug candidates that are normally believed unpromising to stay away from suspension or abortion.

To ameliorate the oral bioavailability, various lipid nanocarriers have been explored for oral delivery of poorly water-soluble drugs. For instance, Wang et al. utilized SLNs to orally delivery sorafenib, an anticancer agent for hepatocarcinoma, in order to achieve a desirable liver targeting [173]. The designed sorafenib-loaded SLNs possessed a smaller particle size and high entrapment efficiency and resulted in enhancement of drug selectivity and bioavailability after oral administration compared with its suspensions. In our group, we developed different kinds of NLCs for the oral delivery of oridonin and tripterine, two natural active ingredients. A biotin-modified NLCs formulation was first investigated for its performance in bioavailability enhancement of oridonin [174]. Compared with the common NLCs, biotin-modified NLCs apparently improved the absorption rate of oridonin rather than absorption extent. However, both biotinylated NLCs and common NLCs significantly enhanced the bioavailability of oridonin relative to the suspensions formulation. The second typical example is the broccoli lipid-based NLCs for the oral delivery of tripterine. We extracted the lipidic components from broccoli using 1-octanol as solvent in an attempt to further the intestinal permeability of NLCs [175]. The results showed that the intestinal permeability and bioavailability of tripterine were largely improved through such functional NLCs. Nanoemulsions, another representative lipid nanocarrier, have also demonstrated a great potential in promoting oral absorption of poorly water-soluble drugs. Yin et al. developed biocompatible nanoemulsions using hemp oil and less surfactants for the oral delivery of baicalein [93], and Chavez-Zamudio et al. prepared lysophosphatidylcholine-stabilized nanoemulsions for the oral delivery of curcumin [176]. The constructed nanoemulsion systems unexceptionally enhanced the oral bioavailability of payloads in comparison with their coarse dispersions. A nanoemulsion system loading dabigatran etexilate phospholipid complex was also proposed for use to improve the lipophilicity and oral bioavailability of drug [177]. In terms of liposomes, there were also some reports related to their use in the oral delivery of poorly water-soluble drugs [100]. As an example, Rushmi et al. formulated black seed oil (Nigella sativa) into liposomes using the ethanol injection method aiming to enhance the oral bioavailability and improve the therapeutic activity of such analgesic in small animal studies of analgesia [178]. The in vivo studies showed that the liposomal formulation demonstrated a significant analgesic activity in mice. In recent years, phytosomes (phospholipid complexes) are also being widely used for oral drug delivery [103]. For example, Freag et al. developed self-assembled phytosomal nanocarriers for improving the solubility and oral bioavailability of celastrol [151], and Telange et al. developed apigenin-loaded phytosomes to improve the drug’s aqueous solubility, dissolution, in vivo bioavailability, and antioxidant activity [106]. It demonstrates that phytosome technique is a promising and viable formulation strategy for enhancing the delivery efficiency of poorly water-soluble drugs.

Although lipid carriers have been proven to be potential as oral delivery vehicles, it should be noted that the lipolysis of lipid carriers substantially takes place in the gastrointestinal transport process. As a matter of fact, the in vivo degradation of lipid nanoparticles is not contradictory with their absorption-promoting effect, since lipids can facilitate drug absorption by co-transport and cytosis in the form of intact nanoparticles or reconstituted micelles [20]. The in vivo lipolysis-reconstitution mechanism of lipid nanocarriers have been confirmed by Wu’s laboratory utilizing an environment-responsive probe [179,180]. The fact that water-quenched fluorescent dye encapsulated in lipid nanoparticles can be rekindled by the reconstitution of lipolytic products after lipolysis of nanoparticles provides pivotal evidence for the gastrointestinal disposition of lipid nanocarriers.

### 4.5. Translation of Liquid Lipid Dispersions into Solid Formulations

Lipid-based formulations are generally liquid preparations that have low physiochemical stability, which will cause inconveniences for storage and quality control. Although lipid nanocarriers belong to colloidal dispersion system, they are just stable on a short-term basis. When storing for a long time, the phenomena of nanoparticle aggregation and precipitation would inevitably occur due to particle collision and gravitational settling [181]. The stability study for lipid nanoparticles in literature is relatively insufficient, and the investigation period for reserved samples is also short, oftentimes not more than one month. For oral delivery, the physical instability of lipid dispersions is not a serious problem, unless rancidity or contamination takes place in the formulation. However, in aqueous conditions, the lipid excipients and drugs tend to deteriorate and degrade, resulting in harmful chemical molecules. In view of safety and conveniences for storage and use, it is best to solidify the liquid preparations. Despite numerous merits, one long-standing historical challenge for the practical application of lipid nanocarriers remains unmet: redispersibility after drying. How to realize the translation of liquid lipid dispersions into solid formulations has gotten into the research focus of nanomedicine [182].

#### 4.5.1. Freeze Drying

Freeze drying is a practicable technique to settle the long-term storage of colloidal nanoparticles. By removing the water from the aqueous dispersions, a dried form of lipid dispersions is harvested whereby to improve the physiochemical stability of colloidal nanoparticles. Freeze drying of nanosuspensions not only requires consideration for the formulation factors, but also the lyophilization process. The process conditions have a crucial effect on the stability of nanoparticles during and after freeze drying. During lyophilization, the nanoparticles will be subjected to various stresses, such as particles agglomeration and desiccation, which may be detrimental to the stability of nanoparticles. Therefore, a proper cryoprotectant and optimized lyophilization process must be adopted so as to minimize damage to the nanostructures. For example, Howard et al. tested nine kinds of cryoprotectants and different freeze drying conditions to optimize lyophilization process of solid lipid nanoparticles loading dexamethasone palmitate for improving the long-term stability [183]. The resulting lyophilized SLNs exhibited slightly larger but acceptable particle size and polydispersity index. Drug loading and particle shape were well maintained by lyophilization. The lyophilized SLNs possessed a consistent particle size and less drug content loss during a three month period. To improve the dissolution and intestinal permeability of diosmin, Freag et al. utilized the lyophilization technique to prepare diosmin-loaded phytosomes [184]. The lyophilized phytosomal nanocarriers exhibited the lowest particle size to 316 nm, adequate ζ-potential for stabilization of colloidal particles, and good in vitro stability. Phytosomes obtained by freeze drying significantly improved the drug’s dissolution and permeation characteristics. Freeze drying is proven to be an effective means to achieve a long-term stability of lipid nanocarriers.

#### 4.5.2. Spray Drying

Spray drying is proposed for use as a promising alternative to stabilize and preserve the colloidal nanoparticles in a dried form. In spraying drying, the liquid is promptly evaporated when the liquid lipid dispersions are sprayed into the hot air stream, in which solid micropowders such as starch and aerosil or blank pellets are pre-charged. The nanoparticles immediately precipitate on the surface of carriers upon water evaporation. This dehydration-solidification technique intended for lipid nanoparticles is normally termed as nano spray drying [185,186]. It is worth noting that spray drying of lipid nanoparticles must prevent particle adhesion, coalescence, and fusion. It requires the spray speed, temperature and carrier excipients to be of optimal conditions. By controlling the processing parameters precisely, the maintenance of colloidal characteristics of nanoparticles can be substantiated through spray drying. For instance, Tian et al. investigated and characterized solidification of nanostructured lipid carriers (NLCs) onto pellets by spray drying using a fluid-bed [187]. To achieve good coating and redispersibility of nanoparticles, PVP k17 was used as the carrier dispersant to load the solidified NLCs. It was found that reconstituted NLCs had spherical morphology similar to the original modality, but had an augmented particle size. Nevertheless, both solidified NLCs and original NLCs showed parallel in vitro lipolysis profiles and pharmacokinetics in beagle dogs. The study indicates that spray drying is a practicable approach to solidifying lipid nanoparticles [188].

#### 4.5.3. Self-Emulsifying

Self-emulsifying drug delivery systems (SEDDS) are emulsifiable water-free formulations made up of oil and emulsifier with or without hydrophilic co-solvent. SEDDS are utilized to solve low bioavailability issues of poorly soluble and highly permeable drugs [189]. Self-emulsifying formulations can rapidly disperse in the gastrointestinal tract in contact with the digestive fluids under the agitation of gastrointestinal peristalsis to spontaneously form nanoemulsions, so-called *in situ* self-emulsification. SEDDS are physically stable formulations and can be manufactured into the semisolid soft capsules (e.g., Sandimmun^®^) [190] or liquisolid tablets [191]. Water-free formulations offer an opportunity to realize the commercial success of nanoemulsions.

#### 4.5.4. Developing Liquisolid Hybrid Formulations

This approach converts liquid lipid nanoparticles into powdered form and then formulates them into tablets or capsules, which differs from the following liquisolid system directly developed from a drug solution. The liquid lipid dispersions must be concentrated to a relatively small volume that can be fully adsorbed by additional adjuvants, such as microcrystalline cellulose (MCC) and aerosil. Liquisolid compact tablets or capsules can be obtained after lipid dispersions are loaded into adsorptive excipients and form dry-looking, freely flowing and compressible powders. For example, Nnamani et al. developed low-dose liquisolid tablets of artemether-lumefantrine (AL) from NLCs and estimated their potential for oral delivery of AL in malariogenic Wistar mice [192]. The results highlighted that AL-loaded NLCs could be further processed into oral tablets to improve the patient’s compliance. 

## 5. Liquisolid Dispersion Technique

### 5.1. Overview of Liquisolid System

**Figure 5:**
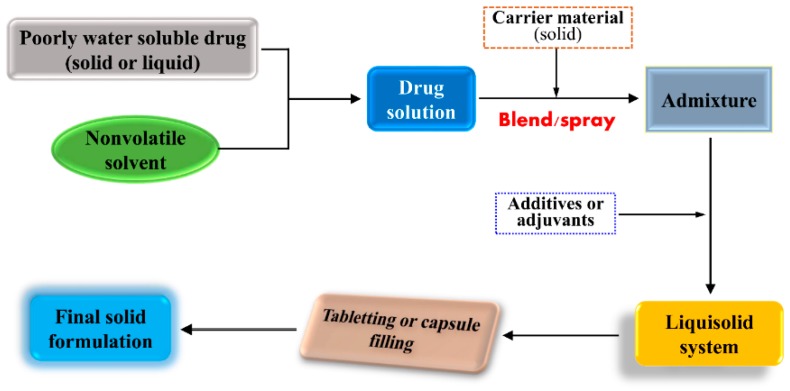
Formulation strategy of poorly water soluble drugs using the liquisolid dispersion technique.

Molecular solution represents the highest dispersion of drug in a variety of formulations. In SDs, a poorly water soluble drug can form solid solution with a suitable carrier excipient. Solid pharmaceutical intermediates are readily processed into a final dosage form, such as tablets and capsules. Accordingly, there should be a possibility by which liquid substances can be changed into the solid form. Liquisolid dispersion technique is the right means to materialize solidification of liquefied drug. A liquisolid system refers to formulation implemented by conversion of lipophilic drug in a nonvolatile solvent into dry-looking, freely flowing and compressible powders by blending a drug solution with adsorptive excipients followed by processing into suitable dosage forms (as illustrated in Figure 5) [193]. The liquisolid system greatly maintains the molecular dispersion of candidate drug and provides the most favorable dissolution condition, freely diffusing into the dissolution medium. Therefore, the liquisolid system possesses a number of advantages: (1) drug dispersion in the solid formulation as solubilized liquid form; (2) enabling solidification of liquid drug; (3) quicker drug dissolution from formulations due to superior wettability and miscibility; (4) low cost of production; and (5) liquisolid dispersions able to be developed either into immediately-release or into sustained-release preparations. Besides these advantages, it requires the dose of drug for developing a liquisolid system to be relatively low in order that the drug solution can be fully loaded within the solid carriers. Liquisolid dispersion technique is becoming an innovative and promising formulation strategy that can improve the dissolution and bioavailability of poorly water soluble drugs [194]. Liquisolid dispersion technique is mainly implemented to enable the formulation of amphiphobic drugs. These drugs tend to precipitate from carriers, even though they are temporarily entrapped in nanoparticles. 

### 5.2. Formulation Components of Liquisolid System

Liquisolid dispersion system is developed based on the principle of converting liquid medication into freely flowing, readily compressible and apparently dry powders by physical adsorption using selected excipients. In addition to common excipients involved in tablets or capsules, a liquisolid dispersion system generally consists of non-volatile solvent, carrier material and coating material. The formulation composition of a typical liquisolid system (e.g., liquisolid compact tablets) is generalized in Table 3.

Non-volatile solvent used in liquisolid systems should be inert, lowly viscous, preferably water-miscible and meanwhile have a strong solvent power as liquid vehicle. Various solvents with high boiling point are explored for the formulation of liquisolid systems, including PEG, propylene glycol, glycerin, and polysorbate. The non-volatile solvent simultaneously acts as a solvent and a binding agent in the liquisolid formulation. It has been shown that the solvent had a significant effect on drug release from the liquisolid system [211]. For rapid-release purpose, a liquid vehicle in which the API is most soluble is normally selected. In the case of sustained-release preparation, the solvent with a highly viscosity is usually used to dissolve the drug, such as glycerin and Cremophor^®^ EL. Of course, other hydrophilic or amphipathic solvents such as Transcutol^®^, Solutol^®^ and Labrasol^®^ are also oftentimes involved in the liquisolid systems. 

In the liquisolid system, carrier materials play the key role in gaining the dry form of powders from the solubilized liquid drug. Carrier materials mainly contribute to liquid adsorption. It requires that the carrier should be porous and possess large enough specific surface area (SSA) [212]. Carrier selection depends on its liquid adsorption capacity, flowability of powders and compressibility. The most commonly used carriers in liquisolid formulations include MCC (e.g., Avicel^®^ and Ceolus^®^), lactose, sorbitol, anhydrous dibasic calcium phosphate (Fujicalin^®^), amorphous magnesium aluminometasilicate (Neusilin^®^), etc. Among these, Fujicalin^®^ and Neusilin^®^ represent the newly-developed carrier materials, which exhibit especially large SSA, up to 40 m^2^/g and 300 m^2^/g, respectively [210]. Neusilin^®^ not only can be used as a carrier material, but also function as a coating material by virtue of excellent adsorption and flowability properties [204].

**Figure 6:**
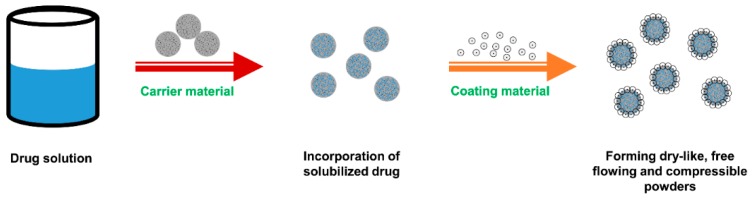
Formation process of dry-looking, freely flowing and compressible powders in the liquisolid system.

Coating material plays a key role in covering the wet carrier particles to form dry-looking and freely flowing powders. It should be a material possessing fine and highly adsorptive micropowders that can adsorb excess liquid to ensure good flowability of the admixture. In the liquisolid system, coating material and carrier material must be used in conjunction in order to enhance the flowing and compressing properties of powders as illustrated in Figure 6. Currently, the most commonly used coating material in the liquisolid formulation is colloidal silicon dioxide, such as Aerosil^®^ and Cab-O-Sil^®^ M5. Amorphous silica gel (e.g., Syloid^®^), Neusilin^®^, calcium silicate (Florite^®^) and ordered mesoporous silicates that have suitable flowability and compressibility can also be used to prepare liquisolid formulation [11].

Apart from non-volatile solvent, carrier and coating materials, the liquisolid system usually use some other additives to develop solid dosage forms. Tablets or capsules normally experience the disintegration process before dissolution. Therefore, disintegrants such as sodium starch glycolate, croscarmellose sodium, and low-substituted hydroxypropyl cellulose (L-HPC) are generally included in liquisolid formulation to allow a fast disintegration. In addition, release modifier (e.g., HPMC) and crystal growth inhibitor (e.g., PVP) are frequently contained in the liquisolid compact tablets [203,213]. These additives improve the dissolution profile and physical stability of liquisolid systems.

### 5.3. Preparation of Liquisolid Compacts

A given powder can only retain limited amount of liquid medication while maintaining acceptable flowability and compressibility. Before preparation, it generally performs the pre-formulation studies, including solubility study, determining the flowable liquid retention potential (Φ-value), calculating the liquid load factor (Lf), and measuring the repose angle of admixture. Theory on liquisolid system was developed in 1992 by Spireas and his colleagues based on the concept of powdered solution [214].

According to the pre-formulation’s information, the candidate drug is dissolved in the required quantity of nonvolatile solvent, and then the resulting drug solution is incorporated into the calculated amounts of carrier and coating materials. Generally, the mixing process is carried out in three steps as proposed by Spireas and Bolton [215]. During the first stage, the liquid medication and carrier material were mixed using a blender at a speed of 60 rpm for 1 min around in order to evenly distribute the liquid mixture in the powders. Afterwards, the coating material in calculated amount is added and blended homogeneously. In the second stage, the resulting admixture was evenly spread as a uniform layer on the surfaces of a mortar and left standing for approximately 5 min to facilitate a complete adsorption of liquid medication by the powder particles. In the third stage, the powders were scraped off from the mortar surface and then blended with a suitable disintegrant at a higher rate for another several minutes. Finally, the prepared liquisolid system that has been subjected to critical evaluation for flowability and compressibility can be further compressed or encapsulated into a specific solid dosage form [216]. 

### 5.4. Liquisolid System-Based Enhancement of Dissolution and Bioavailability

Liquisolid dispersion technique has been widely used to improve the dissolution and bioavailability of poorly water-soluble drugs with a low dose [210]. A liquisolid formulation allows an insoluble drug to be solubilized, almost molecularly dispersed in a solid dosage form, which greatly enhances the dissolution rate of solubility-limiting drugs due to ameliorative wetting property and dissolution surface area, hence the oral bioavailability. There a great number of poorly water-soluble drugs have been developed into liquisolid formulations in an attempt to enhance their dissolution and/or bioavailability, including Rosuvastatin [195], Aprepitant [217], Fenofibrate [218], Curcumin [198], Indomethacin [219], Griseofulvin [205], Loperamide [208], Ketoprofen [220], Olmesartan Medoxomil [207], Loratadine [221], Nimesulide [222], Furosemide [223], Lovastatin [224], Carbamazepine [203], Telmisartan [200], Valsartan [225], and Mosapride Citrate [196]. 

Sharma et al. developed a liquisolid system to improve the dissolution rate and bioavailability of curcumin using PEG as liquid vehicle, Avicel^®^ PH102 as carrier material and Aerosil^®^ as coating material [198]. The systems were screened for pre-compression properties before being compressed to liquisolid tablets, followed by post-compression tests and in vitro dissolution. The optimized formulation exhibited a higher accumulative drug release than directly compressed tablets. Ex vivo permeation of curcumin was significantly enhanced, and the oral bioavailability was increased 18.6-fold in New Zealand rabbits. The authors concluded that solubility promotion of curcumin in liquisolid tablets contributed significant enhancement to its permeation and bioavailability. Fexofenadine hydrochloride (FXD) possesses poor water solubility and pharmacokinetic property. To increase its oral bioavailability and shorten the time to reach the maximum plasma concentration, Yehia et al. formulated FXD into liquisolid tablets with propylene glycol or Cremophor^®^ EL, Avicel^®^ PH102 and Aerosil^®^ 200 as functional excipients [199]. It was found that Cremophor-based liquisolid powders showed acceptable to good flow property suitable for compaction. The physicochemical properties and disintegration time were appropriate for tablet qualities. Dissolution of prepared tablets could be completed within 5 min, and the oral bioavailability of FXD was enhanced by 62% relative to commercial tablets (Allegra^®^). 

In another work, Khames et al. developed risperidone liquisolid formulation using versatile solvents (Transcutol HP, Labrasol and Labrasol/Labrafil (1:1) mixture) as liquid vehicles and evaluated its dissolution and bioavailability [202]. The results showed that liquisolid tablets prepared using Labrasol/Labrafil (1:1) mixture as liquid vehicle with 10% risperidone was a compatible formula with low drug crystallinity and higher dissolution rate (100% in 25 min). The oral bioavailability of risperidone was significantly enhanced through liquisolid tablets in comparison to the conventional tablets (1441.711 μg·h/mL vs. 321.011 μg·h/mL in *AUC*). The examples above indicate that liquisolid technique was a potential tool in increasing dissolution and bioavailability of poorly water-soluble drugs. 

## 6. Other Dispersion Techniques

### 6.1. Co-Precipitation Technique

Co-precipitation technique refers to a process by dissolving a drug in a solvent containing an insoluble adsorbing material and then evaporating them to result in drug precipitation onto the surface or the internal pore of the absorbing material, thus forming solid dispersions [226]. It is different from the solvent evaporation method used for preparation of SDs. In this process, the drug is completely dissolved in a volatile solvent, but the carrier material is suspended in the selected solvent. The carrier materials are generally water insoluble and possess strong adsorptive capacity and high porosity. The commonly used carrier materials include crospovidone (PVPP), sodium starch glycolate (CMS-Na), L-HPC, mesoporous silica, mesoporous carbons, etc. Upon the solvent evaporating, the drug precipitates onto the carrier excipient to form drug/excipient dispersions. The viscosity of dispersions produced by such technique is lower than that prepared by the solvent evaporation method, but the drug dispersibility is lower than the latter. Shin et al. prepared coprecipitates of furosemide and crospovidone by a solvent evaporation method [227]. The dissolution rate of furosemide was markedly enhanced by coprecipitating with crospovidone. They confirmed that physicochemical modifications at the molecular level have taken place between furosemide and crospovidone in the coprecipitates that changed the thermal property and increased the dissolution of furosemide. Planinsek et al. prepared carvedilol/porous silica dispersions from acetone solution through evaporating the solvent to cause drug precipitation and adsorption into the pores of silica [228]. The dispersions resulted in a significant improvement in drug dissolution compared with raw material and its physical mixture. It was shown that amorphous form of carvedilol in the dispersions, improved wettability and weak interactions between the drug and carrier contributed to dissolution enhancement.

### 6.2. Concomitant Crystallization Technique

In recent years, pharmaceutical cocrystals have gained increasing attention to developing drug in solid-state formulations [229], which can be achieved through a concomitant crystallization technique. Cocrystal complexes structurally contain drug candidate and co-crystallizing agent (coformer). Cocrystals are formed by intermolecular interactions or synthons between the drug and co-crystal former, which results in the creation of supramolecular assembly. The physiochemical properties of drug depends on its molecular order in the solid form, and changes in intermolecular interactions have important effects on these properties, including melting point, stability, solubility and dissolution. From the micromilleu of drug being, co-crystals can be viewed as a kind of pharmaceutical dispersions. Although the drug is always crystallized in the cocrystals, the physiochemical properties can be greatly modified through developing cocrystals. For poorly soluble drugs, the most predominant changes in cocrystal system refer to its apparent solubility and dissolution improvement [230]. Pharmaceutical cocrystals can be manufactured by two basic methods: (1) solvent-free technique (grinding, solvent-assisted grinding and sonication) and (2) solvent-based technique (slurring, solvent evaporation, and antisolvent cocrystallization). For example, Du et al. successfully obtained two novel cocrystals of lamotrigine with 4,4′-bipyridine and 2,2′-bipyridine as coformer by neat grinding and liquid assisted grinding [231]. The resulting cocrystals exhibited significantly improved solubility and dissolution rate in comparison with the monocrystal drug. In another case, a highly soluble carbamazepine (CBZ) cocrystal with glutaric acid (GLA) was developed by Yamashita and Sun through solvent evaporation [232]. The dissolution rate of CBZ was improved in the case of CBZ/GLA cocrystals due to solubility increase and precipitation inhibition during dissolution. Pharmaceutical co-crystals provide new opportunity to modify the physicochemical properties of poorly water-soluble drugs whereby to enhance their dissolution rate and bioavailability. 

### 6.3. Inclusion Complexation Technique

In most cases, inclusion complexes are not regarded as a pharmaceutical dispersion. However, it is unreasonable to exclude inclusion complexes from pharmaceutical dispersions, since the guest molecule (drug) is fully incorporated into the cavity of host molecule that results in a complete dispersion of drug. It is merely that, in solid dispersions, the drug is embedded between carrier molecules, but in inclusion complexes, the drug is embedded within the molecule. In addition, solid dispersions and cyclodextrin inclusion complexes shared some common preparative techniques, such as kneading method and solvent evaporation method. From these points of view, drug inclusion into another molecule can be deemed as a practicable dispersion technique. Cyclodextrin complexation, but not limited to cyclodextrins, has shown the potential to precisely improve the aqueous solubility, dissolution rate, and bioavailability of poorly water-soluble drugs [233]. For instance, Ezawa et al. prepared piperine/cyclodextrin inclusion complexes by the co-grinding method and tested their solubility and dissolution [7]. They found that piperine could form inclusion complexes with α-cyclodextrin and γ-cyclodextrin in a stoichiometric ratio of 1/2 and 1/1, respectively, and the complexes of piperine/α-cyclodextrin exhibited higher solubility than that of piperine/γ-cyclodextrin. Mady et al. utilized a coprecipitation method to investigate the complexing effect of finasteride, a BCS II drug, with different kinds of β-cyclodextrin derivatives [234]. It was found that the dimethyl-β-cyclodextrin (DM-β-CYD) inclusion system gave rise to the highest complexation efficiency for solubility improvement and hence the bioavailability. These cases indicate that dispersion resulting from inclusion complexation can act as the enhancement of pharmacokinetics of poorly water-soluble drugs [235].

## 7. Conclusions

To date, oral administration remains the most preferred route that patients take the medicine, and solid dosage forms are the prevalent modality of pharmaceutical preparations. Pharmaceutical processing always results in a dispersion of a drug in the solid, semisolid or liquid excipient in particulate, colloidal, amorphous or molecular state. Super-advanced dispersion can engender additional benefits for dissolution and bioavailability of poorly soluble drugs, which, of course, greatly depends on effective dispersion techniques. It has been shown that solid dispersion, lipid-based dispersion and liquisolid dispersion are becoming useful tools to solve the formulation challenges of poorly soluble drugs. 

## Figures and Tables

**Table 1:** Summary of the commonly used excipients for preparation of solid dispersions.

Carrier Excipients	Example	Comments	Reference
Saccharides	Sucrose Glucose Lactose Dextrose	Ordinary dispersibility; having potential effect on drug absorption.	[29,30,31,32,33]
Alcohols	Mannitol Sorbitol	Ordinary dispersibility; weak absorption-promoting effect.	[34,35] [36]
Organic acids	Citric acid Tartaric acid	Effervescent dispersion;Simple dispersing material, not applicable for acid-labile API.	[37,38] [39]
Polyethylene glycol	PEG 4000 PEG 6000	High dispersibility; able to solubilize drug and delay aging of SDs.	[40] [41,42]
Polyvidone	PVP k15 PVP k30	High dispersibility; able to inhibit recrystallization.	[42] [23,43,44]
Cellulose derivative	HPMC HPC MC	High dispersibility; less plasticity and hygroscopicity, easy to process.	[45,46] [47,48] [49]
Poly(oxyethylene–*co*-oxypropylene)	Poloxamer 188 Poloxamer 407	High dispersibility; able to solubilize drug and having absorption-promoting effect.	[50,51] [52]
Carboxypolymethylene	Carbopol 947 Carbopol 907	Ionic polymers; good dispersibility; rapid drug release in the intestine.	[53] [54]
Polyoxyethylene stearate	Polyoxyethylene (40) stearate	Fine dispersibility; contribute less to dissolution; used rarely.	[55]
Fatty acid macrogolglycerides	Gelucire 44/14 Gelucire 50/13	Functional dispersing materials; either able to enhance dissolution or to promote drug absorption.	[56,57] [58,59]
Poly(vinylpyrrolidone-*co*-vinyl acetate)	PVP/VA	Fine dispersibility but low hygroscopicity; superior to PVP in function.	[60,61]
Poly(vinyl acetate-*co*-vinyl caprolactame-*co*-ethylene glycol)	Soluplus^®^	Novel dispersing material; excellent capability to form solid solution.	[62,63]

**Table 2:** Commonly used lipid excipients in lipid-based nanocarriers.

Lipid Excipient	Chemical	Carrier Type	Comments	Reference
Soybean oil	Long-chain triglycerides	Nanoemulsions; NLCs	Liquid, high biocompatibility, negligible physiological effect, solubilizing capacity a little weak.	[109,110,111,112]
Olive oil	Long-chain triglycerides	Nanoemulsions; NLCs	Liquid, good health benefits, containing more monounsaturated fatty acid, easy to emulsify.	[110,113,114,115,116]
Hemp oil	Medium/long-chain triglycerides blended with low-molecular-weight lipids	Nanoemulsions	Liquid; rich in essential fatty acids, having tocopherols, tocotrienols, phytosterols, phospholipids, etc., excellent hydrophilicity and self-emulsifiability.	[93,117]
Caprylic/capric triglycerides	Medium-chain triglycerides	Nanoemulsions; NLCs	Liquid, fine solubilizing capacity, good compatibility with other lipids, easy to emulsify.	[118,119,120,121,122,123]
Captex^®^ series	Medium/short-chain triglycerides	Nanoemulsions; SEDDS; NLCs	liquid, fine solubilizing and emulsifying capacities, miscible with other lipids.	[124,125,126]
Capmul MCM	Medium-chain mono/diglycerides	Nanoemulsions; SEDDS; NLCs	Liquid, excellent solvent powder for many organic compounds, can use as emulsifier.	[127,128,129,130]
Capmul MCM C8	Glyceryl monocaprylate	Nanoemulsions; SEDDS; NLCs	Liquid, properties similar to that of Capmul MCM.	[131,132,133]
Maisine ^TM^ 35-1	Glyceryl monolinoleate	SEDDS	Liquid, solubilizer, bioavailability enhancer, oil phase in SEDDS.	[134,135,136,137]
Peceol^TM^	Glyceryl monooleate	SEDDS; NLCs; Cubosomes;	Liquid, lipid dispersion agent, oil-soluble surfactant, moisturizer.	[138,139,140]
Lauroglycol^®^ 90	Propylene glycol monolaurate	Nanoemulsions; SEDDS; NLCs	Liquid, water insoluble surfactant of SEDDS, solubilizer, bioavailability enhancer, skin penetration solubilizer enhancer.	[141,142,143]
Capryol^TM^ series	Propylene glycol monocaprylate	Nanoemulsions; SEDDS; NLCs	Liquid, properties similar to that of Lauroglycol^®^ 90.	[144,145,146]
Labrafil M 1944 CS	Oleoyl polyoxyl-6 glycerides	Nanoemulsions; SEDDS; NLCs	Liquid, water dispersible surfactant, able to self-emulsify, good miscibility with other lipids, bioavailability enhancer, solubilizer, co-emulsifier.	[147,148,149]
Lecithin	Phosphatidylcholine blended with a small amount of other lipid components.	Liposomes; phytosomes; sorts of lipid nanoparticles	Semi-solid, an amphiphilic lipid, used as vesicles-forming material, solubilizing, emulsifying, and stabilizing agents.	[150,151,152,153,154]
Gelucire^®^ series	Lipid blends consisting of mono-, di-, or triglycerides and fatty acid macrogolglycerides	SEDDS; SLNs; NLCs	Semi-solid, non-ionic water soluble surfactant for solid/semi-solid dispersions and SEDDS, bioavailability enhancer, micelles-forming material, solubilizing and wetting agent.	[146,155,156]
Monostearin	Glyceryl monostearate	SLNs; NLCs	Solid, lipid matrix for SLNs and NLCs; thickening, solidifying and control release adjusting agent.	[133,157]
Precirol^®^ ATO 5	Glyceryl distearate	SLNs; NLCs	Solid, lipid matrix for SLNs and NLCs, hydrophobicity and melting point greater than glyceryl monostearate.	[158,159]
Compritol^®^ 888 ATO	Glyceryl behenate	SLNs; NLCs; solid lipid dispersions	Solid, high melting point lipid, used for preparation of SLNs and NLCs, lipid matrix for sustained release, used as atomized powders.	[160,161,162]
Trilaurin	Glyceryl trilaurate	SLNs; NLCs;	Solid, lipid matrix for SLNs and NLCs, sustained release material, thickening agent.	[163,164,165]
Cetyl palmitate	Palmityl palmitate	SLNs; NLCs;	Solid, wax-like substance, used for preparation of SLNs and NLCs.	[166,167]
Tripalmitin	Glyceryl tripalmitate	SLNs; NLCs;	Solid, as lipid matrix of SLNs and NLCs, skin-conditioning agent.	[168,169]

**Table 3:** Components generally involved in a liquisolid formulation.

Excipients Type	Characteristics	Function	Examples	Reference
Non-volatile solvent	Inert, water-miscible, compatible with drug candidate, excellent dissolving powder.	Non-volatile solvent acts as a solvent and a binding agent in a liquisolid system.	PEG series; glycerin; propylene glycol; polysorbate; Cremophor^®^ EL; Transcutol HP; Capryol^TM^ 90; 2-pyrrolidone; Labrasol, etc.	[195,196,197,198,199,200,201,202]
Carrier material	Porous, large specific surface area, sufficient adsorption ability, good flowability and compressibility.	Carrier material plays a fundamental role in forming the dry form of powders from liquid medication.	Microcrystalline cellulose (MCC, e.g., Avicel^®^, Ceolus^®^, Vivapur^®^, Emcocel^®^); lactose; mannitol; Magnesium Aluminometasilicate (Neusilin^®^); Dibasic calcium phosphate anhydrous (Fujculin^®^);	[196,197,203,204,205,206,207]
Coating material	Ultrafine and highly adsorptive particles, good flow-aided effect.	Coating material contributes to covering the wet surface of particles by adsorbing excess liquid to ensure a good flowability of powders.	Colloidal silicon dioxide (e.g., Aerosil^®^, Cab-O-Sil^®^ M5); Neusilin^®^; Calcium Silicate (Florite^®^)	[195,196,204,208,209]
Other adjuvants	Disintegrant, lubricant, release modifiers, flavoring and coloring agents, etc.	The selected adjuvants can improve the quality of solid dosage forms.	Sodium starch glycolate (CMS-Na); crospovidone; L-HPC; PVP k25; PEG 6000; HPMC; Eudragit.	[22,210]
